# Report of a Misleading Case of the Superior Mesenteric Artery Syndrome

**DOI:** 10.1155/2022/6013579

**Published:** 2022-05-27

**Authors:** Amir Mohammad Salehi, Hossain Salehi, Maryam Hasanzarrini, Elham Khanlarzadeh

**Affiliations:** ^1^School of Medicine, Hamadan University of Medical Sciences, Hamadan, Iran; ^2^Gastroenterology Ward, Farhikhtegan Hospital, Azad University of Medical Sciences, Tehran, Iran; ^3^Hepatology of Hamadan University of Medical School, Hamadan, Iran; ^4^Department of Community Medicine, School of Medicine Hamadan University of Medical Sciences, Hamadan, Iran

## Abstract

Superior mesenteric artery syndrome (SMAS), which is also known as the cast syndrome, Wilkie's syndrome, or chronic duodenal ileus, is a specific type of duodenal obstruction characterized by the obstruction of the inferior part of the duodenum due to its compression between the superior mesenteric artery (SMA) and the aorta. This problem is usually resulting from loss of the mesenteric fat pad. The present report describes a case of SMAS who was an 18-year-old woman presenting with weight loss and postprandial pain. The patient was initially diagnosed with *Helicobacter pylori* infection and underwent antibiotic therapy. However, the related symptoms did not resolve. Finally, she was ordered a CT scan, which led to the diagnosis of SMAS.

## 1. Introduction

Superior mesenteric artery syndrome (SMAS) was first described in 1861 by Carl von Rokitansky. Afterward, Wilkie spelled out its detailed clinical and pathophysiological characteristics, naming the condition as the chronic duodenal ileus [1]. SMAS is a problem of the upper gastrointestinal (GI) tract resulting from a life-threatening duodenal compression that mainly presents itself with rapid and significant weight loss, with the most prominent symptoms including early satiety, postprandial pain, and bile reflux [2]. The diagnosis is usually challenging, particularly in young patients and infants. The suitable approach for SMAS diagnosis is controversial because the symptoms are not always relevant or consistent with the radiological findings. Moreover, in several cases, symptoms are not fully resolved after the treatment [1]. Therefore, the problem is usually underdiagnosed or misdiagnosed with other anatomical or motility-related causes of the duodenal obstruction [3].

## 2. Case Report

Our case report describes an 18-year-old female patient who presented to our clinic about 6 months ago with severe epigastric pain, nausea, heartburn, regurgitation, and weight loss (about 5 kg). Her epigastric pain had a colic nature and did not radiate to other parts of the abdomen, the regurgitation content included bile secretions, and there was no report of dysphagia. Moreover, she had no history of malignancy, trauma, burn, malabsorption, spinal cord injury, neurological diseases, or long-term hospitalization. No significant finding was reported in the physical examination except abdominal distention without any evidence of tenderness or hyperactive bowel sounds. The patients underwent endoscopy, revealing antral erythema and mild to moderate gastritis. Eventually, she was diagnosed with *Helicobacter pylori* infection using gastric biopsy and was prescribed a one-month course of antibiotic therapy.

**Figure 1:**
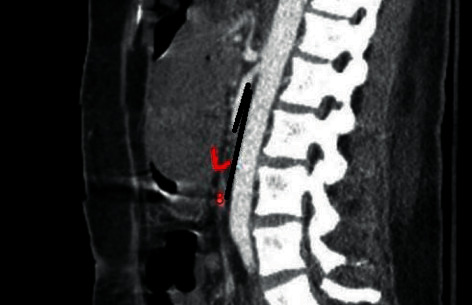
CT sagittal view showing an angle of 8 degrees between the superior mesenteric artery and aorta.

**Figure 2:**
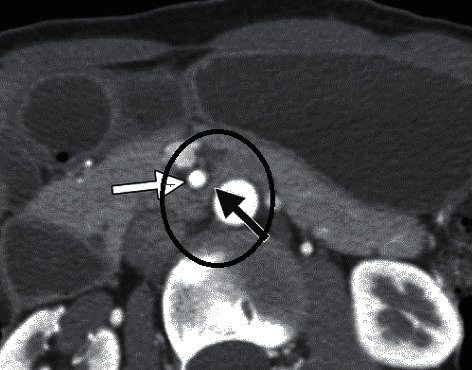
CT scan (axial view) with duodenal compression (black arrow) between the aorta and superior mesenteric artery (white arrows).

However, there was no symptom alleviation. After one month, the patient presented to our clinic for the second time with the same symptoms. Therefore, she was rehospitalized and underwent abdominal and pelvic CT scan with IV contrast (Figures 1 and 2). According to the CT findings, the proximal part of the duodenum was dilated, and the angle between the aorta and SMA was about 8 degrees. A consultation with the surgery service was ordered for the patient to confirm the diagnosis of SMAS. Due to the severity of epigastric pain and severe reduction of the angle between the aorta and SMA, the patient was then transferred to the surgery ward and underwent laparoscopic duodenojejunostomy. Following abdominal insufflation, a laparoscope was introduced into the peritoneal cavity, which showed the dilation of the proximal duodenum. However, the jejunum seemed to be normal. Afterward, 3 additional trocars were placed. After retraction of the transverse colon, the Treitz ligament was identified, and SMA was noted to be prominent and lying across the distal duodenum, which was collapsed. A duodenojejunostomy was performed. Then, a side-to-side anastomosis was made. Moreover, the methylene blue test did not show any leakage. The patient responded to the surgery dramatically. Four days after the surgery, she could consume oral fluids and then start a regular diet.

## 3. Discussion

The third part of the duodenum is confined to the mesenteric root and SMA anteriorly, while its posterior part touches the aorta and spine. The angle between the SMA and aorta is about 25–60 degrees, while the duodenum lies in a cavity between these two arteries with a diameter of 10–28 mm. In the SMAS, the angle is reduced to 6–15 degrees, decreasing the cavity diameter to 2–8 mm. Therefore, the descending duodenum appears dilated in the CT scan and is compressed between the aorta and SMA. Other common causes of the SMAS include ectopic origin of the SMA, superior and abnormal location of the Treitz ligament, the presence of a short Treitz ligament, and loss of the retroperitoneal fat, which usually surrounds the SMA and aorta like a cushion [1, 4, 5]. Up to now, about 400 cases of SMAS have been reported [2], with a higher prevalence in women compared to men [3]. According to the studies, the symptoms are usually developed in the acute form of the disease, which can be observed in 0.13%–0.3% of the barium series studies of the upper GI [1].

Most cases of SMAS are associated with severe, debilitating diseases leading to excessive weight loss, such as malignancies, malabsorption syndromes, AIDS [6], trauma, burn [5, 7], bariatric surgery [8], spinal cord injury, paraplegia [9], substance abuse [10], and anorexia nervosa [11], while the syndrome has also been reported in the conditions leading to mild weight loss, such as reconstructive surgery for scoliosis treatment [12], celiac axis compression syndrome [13], and nutcracker syndrome [14].

The symptom severity depends on the underlying cause and obstruction severity. In mild cases, only postprandial pain and early satiety may be reported, while patients with severe SMAS may develop nausea, weight loss, and the symptoms of bile reflux. It is usually believed that the symptoms may resolve when a given patient lies in the lateral decubitus or prone positions as these positions remove the pressure from the mesentery and SMA, widening the space between the SMA and aorta. However, the present patient did not report this phenomenon [1]. The physical examination findings are usually nonspecific and include abdominal distension, succussion splash, and increased bowel sounds. Laboratory findings can be nonspecific as well. However, the patients experiencing severe vomiting or regurgitation may develop significant electrolyte abnormalities, including metabolic alkalosis [15]. Delayed diagnosis of the problem may lead to dangerous complications, such as gastrointestinal perforation, gastric pneumatosis, portal venous gas, formation of a bezoar caused by the duodenal obstruction, electrolyte abnormalities, or deaths [15, 16].

Conservative treatment includes nasogastric tube placement to alleviate the duodenal pressure and treating the electrolyte abnormalities, such as metabolic alkalosis. In patients suffering from severe obstruction, nutritional support using a nasojejunal feeding tube can be necessary to help the patient gain weight [17]. In adult patients with mild disease and children whose disease is probable to be acute, the conservative treatment is usually successful if combined with nutritional support. However, nutritional support is not usually sufficient in patients with chronic disease. These patients should undergo electrolyte abnormality resolution and feeding using a nasogastric tube for a short time. If the symptoms are not resolved, various surgical options should be considered [7, 17, 18], including duodenojejunostomy, gastrojejunostomy, and the Strong's procedure, where the Treitz ligament is divided to free the duodenum from the aortomesenteric space. The preferred surgical option is the duodenojejunostomy, which is performed using the transabdominal or laparoscopic approaches. However, the laparoscopic procedure has recently replaced the transabdominal approach [3]. This procedure is the treatment of choice due to its high chance of success and low possibility of complications, such as ulcers [19].

## References

[1] Singal R., Sahu P., Goel M. (2010). Superior mesenteric artery syndrome: a case report. *North American Journal of Medical Sciences*.

[2] Shukla R. C., Pathak R. (2008). Superior mesenteric artery syndrome: case report. *Nepal Medical College Journal*.

[3] Van Horne N., Jackson J. P. (2020). *Superior Mesenteric Artery Syndrome*.

[4] Lippl F., Hannig C., WeiB W., Allescher H. D., Classen M., Kurjak M. (2002). Superior mesenteric artery syndrome: diagnosis and treatment from the gastroenterologist’s view. *Journal of Gastroenterology*.

[5] Gebhart T. (2015). Superior mesenteric artery syndrome. *Gastroenterology Nursing*.

[6] Stümpfle R., Wright A., Walsh J. (2003). Superior mesenteric artery syndrome in an HIV positive patient. *Sexually Transmitted Infections*.

[7] Reckler J. M., Bruck H. M., Munster A. M., Curreri P. W., Pruitt B. A. (1972). Superior mesenteric artery syndrome as a consequence of burn injury. *The Journal of Trauma, Injury, Infection, and Critical Care*.

[8] Goitein D., Gagne D. J., Papasavas P. K. (2004). Superior mesenteric artery syndrome after laparoscopic roux-en-y gastric bypass for morbid obesity. *Obesity Surgery*.

[9] Laffont I., Bensmail D., Rech C., Prigent G., Loubert G., Dizien O. (2002). Late superior mesenteric artery syndrome in paraplegia: case report and review. *Spinal Cord*.

[10] Barnes J. B., Lee M. (1996). Superior mesenteric artery syndrome in an intravenous drug abuser after rapid weight loss. *Southern Medical Journal*.

[11] Sours J. A., Vorhaus L. J. (1981). Superior mesenteric artery syndrome in anorexia nervosa: a case report. *American Journal of Psychiatry*.

[12] Zadegan F., Lenoir T., Drain O. (2007). Superior mesenteric artery syndrome following correction of spinal deformity: case report and review of the literature. *Revue de chirurgie orthopedique et reparatrice de l’appareil moteur*.

[13] Tseng C.-K., Su W. B., Lai H. C. (2008). Superior mesenteric artery syndrome caused by celiac axis compression syndrome: a case report and review of the literature. *European Journal of Gastroenterology and Hepatology*.

[14] Inal M., Unal Daphan B., Karadeniz Bilgili M. Y. (2014). Superior mesenteric artery syndrome accompanying with nutcracker syndrome: a case report. *Iranian Red Crescent Medical Journal*.

[15] Lim J. E., Duke G. L., Eachempati S. R. (2003). Superior mesenteric artery syndrome presenting with acute massive gastric dilatation, gastric wall pneumatosis, and portal venous gas. *Surgery*.

[16] Fuhrman M. A., Felig D. M., Tanchel M. E. (2003). Superior mesenteric artery syndrome with obstructing duodenal bezoar. *Gastrointestinal Endoscopy*.

[17] Merrett N. D., Wilson R. B., Cosman P., Biankin A. V. (2009). Superior mesenteric artery syndrome: diagnosis and treatment strategies. *Journal of Gastrointestinal Surgery*.

[18] Biank V., Werlin S. (2006). Superior mesenteric artery syndrome in children: a 20-year experience. *Journal of Pediatric Gastroenterology and Nutrition*.

[19] Makary M. S., Patel A., Aquino A. M., Chamarthi S. K. (2017). Clinical and radiologic considerations for idiopathic superior mesenteric artery syndrome. *Cureus*.

